# Genome Sequence of the Halophilic Bacterium Kangiella spongicola ATCC BAA-2076^T^

**DOI:** 10.1128/MRA.00847-18

**Published:** 2018-07-19

**Authors:** Aria Underriner, Tyler Silverwood, Carolyn Kelley, Kyle S. MacLea

**Affiliations:** a Biomedical Science and Technology Program, Seacoast School of Technology, Exeter, New Hampshire, USA; b Biology Program, University of New Hampshire, Manchester, New Hampshire, USA; c Biotechnology Program, University of New Hampshire, Manchester, New Hampshire, USA; d Department of Life Sciences, University of New Hampshire, Manchester, New Hampshire, USA; University of California, Riverside

## Abstract

The Gram-negative genus Kangiella contains a number of halophilic species that display high levels of iso-branched fatty acids. Kangiella spongicola was isolated from a marine sponge, Chondrilla nucula, from the Florida Keys in the United States. A genome assembly of 2,825,399 bp with a 44.31% G+C content was generated from strain A79^T^ (=ATCC BAA-2076^T^).

## Abstract (precis)

The Gram-negative genus Kangiella contains a number of halophilic species that display high levels of iso-branched fatty acids. Kangiella spongicola was isolated from a marine sponge, Chondrilla nucula, from the Florida Keys in the United States.

## ANNOUNCEMENT

Bacteria within the family Alcanivoraceae and class Gammaproteobacteria include the genus Kangiella. Kangiella is notable for being a newly described genus of halophilic bacteria with an unusually high production of iso-branched fatty acids (1, 2). Kangiella spongicola ATCC BAA-2076^T^ is a member of this genus recently discovered from the marine sponge Chondrilla nucula, collected from the Florida Keys in the United States (2). This organism is a Gram-negative nonmotile rod-shaped bacterium that is able to survive in environments with up to 15% NaCl and a wide range of growth temperatures and pH values. The genome sequence generated for K. spongicola may be a useful data point in the understanding of sponge microbiota in marine environments, especially in combination with the five other members of the genus (1, 3, 4) that have been sequenced thus far (5, 6).

Kangiella spongicola ATCC BAA-2076^T^ was bought in lyophilized form from ATCC (Manassas, VA, USA). It was rehydrated in marine broth 2216 (BD, Franklin Lakes, NJ, USA) and incubated in a shaking incubator at 30°C for 24 h. Inoculated marine broth was spread on marine agar 2216 (BD), incubated at the same temperature, and, from the streak plate, a single colony was selected from which to grow a larger liquid culture for production of genomic DNA (gDNA) using a DNA minikit (Qiagen, Valencia, CA, USA). Pure gDNA was tagged with sequence adapters concurrent with fragmentation using the Nextera library prep kit (Illumina, San Diego, CA, USA). Tagged fragments were then sequenced on an Illumina HiSeq 2500 (Hubbard Center for Genome Studies, Durham, NH, USA) instrument. The generated 250-bp paired-end read sequences were bioinformatically trimmed before assembly using Trimmomatic (7). Paired and trimmed reads were assembled into a draft genome using the default settings of SPAdes version 3.11.1 (8). Small contigs and contaminants were removed, and the 140 remaining contigs were analyzed with the NCBI Prokaryotic Genome Annotation Pipeline (PGAP) process for gene prediction and annotation (9) and found to have a total sequence length of 2,825,399 bp, representing an average coverage of 326×. Its closest relatives, K. koreensis and K. aquimarina, are of similar size at 2.85 Mbp and 2.68 Mbp, respectively. Of the 140 contigs, the bulk of the sequence data were found in four contigs with lengths of 1,220,304 bp, 675,233 bp, 545,949 bp, and 83,637 bp. Of the remaining 136 contigs, all were less than 6,001 bp in length. The *N*_50_ value was 675,233 bp with an *L*_50_ value of 2, as determined by QUAST (10). The G+C content of 44.31% was consistent with the initial report of 44.9% (2). A total of 2,544 genes, 2,460 coding DNA sequences (CDS), 36 pseudogenes, 5 rRNAs, and 38 tRNAs were annotated using NCBI PGAP.

### Data availability.

This Kangiella spongicola ATCC BAA-2076^T^ whole-genome shotgun sequence (WGS) project has been deposited in DDBJ/ENA/GenBank under accession number QICH00000000. The version described in this paper is the first version, QICH01000000.

## References

[1] Yoon J-H, Oh T-K, Park Y-H 2004 *Kangiella koreensis* gen. nov., sp. nov. and *Kangiella aquimarina* sp. nov., isolated from a tidal flat of the Yellow Sea in Korea. Int J Syst Evol Microbiol 54:1829–1835. doi:10.1099/ijs.0.63156-0. 15388751

[2] Ahn J, Park J-W, McConnell JA, Ahn Y-B, Häggblom MM 2011 *Kangiella spongicola* sp. nov., a halophilic marine bacterium isolated from the sponge *Chondrilla nucula*. Int J Syst Evol Microbiol 61:961–964. doi:10.1099/ijs.0.021733-0. 20511465

[3] Yoon J-H, Kang S-J, Lee S-Y, Lee J-S, Oh T-K 2012 *Kangiella geojedonensis* sp. nov., isolated from seawater. Int J Syst Evol Microbiol 62:511–514. doi:10.1099/ijs.0.029314-0. 21478391

[4] Lee S-Y, Park S, Oh T-K, Yoon J-H 2013 *Kangiella sediminilitoris* sp. nov., isolated from a tidal flat sediment. Int J Syst Evol Microbiol 63:1001–1006. doi:10.1099/ijs.0.040691-0. 22685109

[5] Han C, Sikorski J, Lapidus A, Nolan M, Glavina Del Rio T, Tice H, Cheng J-F, Lucas S, Chen F, Copeland A, Ivanova N, Mavromatis K, Ovchinnikova G, Pati A, Bruce D, Goodwin L, Pitluck S, Chen A, Palaniappan K, Land M, Hauser L, Chang Y-J, Jeffries CD, Chain P, Saunders E, Brettin T, Göker M, Tindall BJ, Bristow J, Eisen JA, Markowitz V, Hugenholtz P, Kyrpides NC, Klenk H-P, Detter JC 2009 Complete genome sequence of *Kangiella koreensis* type strain (SW-125T). Stand Genomic Sci 1:226–233. doi:10.4056/sigs.36635. 21304661 PMC3035244

[6] Choe H, Kim S, Oh J, Nasir A, Kim BK, Kim KM 2015 Complete genome of *Kangiella geojedonensis* KCTC 23420^T^, putative evidence for recent genome reduction in marine environments. Mar Genomics 24:215–217. doi:10.1016/j.margen.2015.05.015. 26044616

[7] Bolger AM, Lohse M, Usadel B 2014 Trimmomatic: a flexible trimmer for Illumina sequence data. Bioinformatics 30:2114–2120. doi:10.1093/bioinformatics/btu170. 24695404 PMC4103590

[8] Bankevich A, Nurk S, Antipov D, Gurevich AA, Dvorkin M, Kulikov AS, Lesin VM, Nikolenko SI, Pham S, Prjibelski AD, Pyshkin AV, Sirotkin AV, Vyahhi N, Tesler G, Alekseyev MA, Pevzner PA 2012 SPAdes: a new genome assembly algorithm and its applications to single-cell sequencing. J Comput Biol 19:455–477. doi:10.1089/cmb.2012.0021. 22506599 PMC3342519

[9] Tatusova T, DiCuccio M, Badretdin A, Chetvernin V, Ciufo S, Li W 2013 Prokaryotic Genome Annotation Pipeline. National Center for Biotechnology Information, Bethesda, MD.

[10] Gurevich A, Saveliev V, Vyahhi N, Tesler G 2013 QUAST: quality assessment tool for genome assemblies. Bioinformatics 29:1072–1075. doi:10.1093/bioinformatics/btt086. 23422339 PMC3624806

